# Synergistic Theoretical and Experimental Insights into NH_4_^+^-Enhanced Vanadium Oxide Cathodes for Aqueous Zinc-Ion Batteries

**DOI:** 10.3390/molecules29122834

**Published:** 2024-06-14

**Authors:** He Lin, Jing Xu, Yu Zhang

**Affiliations:** State Key Laboratory of Chemistry and Utilization of Carbon Based Energy Resources, School of Chemistry, Xinjiang University, Urumqi 830017, China; mackim90100@gmail.com (J.X.); cnuo017@gmail.com (Y.Z.)

**Keywords:** zinc-ion batteries, cathode materials, ammonium, vanadium oxide

## Abstract

This study explores the enhancement of aqueous zinc-ion batteries (AZIBs) using ammonium-enhanced vanadium oxide cathodes. Density Functional Theory (DFT) calculations reveal that NH_4_^+^ incorporation into V_6_O_16_ lattices significantly facilitates Zn^2+^ ion diffusion by reducing electrostatic interactions, acting as a structural lubricant. Subsequent experimental validation using (NH_4_)_2_V_6_O_16_ cathodes synthesized via a hydrothermal method corroborates the DFT findings, demonstrating remarkable electrochemical stability with a capacity retention of 90% after 2000 cycles at 5 A g^−1^. These results underscore the potential of NH_4_^+^ in improving the performance and longevity of AZIBs, providing a pathway for sustainable energy storage solutions.

## 1. Introduction

The exhaustion of fossil fuels and their consequential environmental ramifications compel the need for optimizing energy frameworks and advancing the development of sustainable and environmentally benign energy sources, including bioenergy, solar energy, hydrogen energy, and wind energy [1]. Given the intermittent nature of renewable energy sources in terms of energy output, efficient energy storage and transportation systems are imperative for their effective utilization. In this context, electrochemical energy storage devices are essential and are required to meet several critical criteria: high safety, the absence of environmental pollution, high energy and power density, resource availability, and an extended cycle life. In recent years, lithium-ion batteries (LIBs) have gained a predominant position in the rechargeable energy market, attributed to their high energy density [2,3,4,5,6]. However, the scarcity of lithium resources poses a significant limitation. Moreover, the organic electrolytes employed in current commercial LIBs are characterized by their toxicity, flammability, and low ionic conductivity [7,8]. In response to these challenges, research efforts are increasingly directed towards exploring alternative battery technologies utilizing potassium (K^+^) [9] and multivalent ions such as magnesium (Mg^2+^) [10], zinc (Zn^2+^) [11], and aluminum (Al^3+^) [12].

Among the investigated alternatives, aqueous zinc-ion batteries (AZIBs) have demonstrated promising electrochemical properties. Zinc metal, which is abundant and cost-effective to process, can be directly employed as an anode material, significantly reducing the manufacturing costs associated with battery production. Importantly, the redox potential of zinc (−0.763 V vs. Standard Hydrogen Electrodes) and its high theoretical capacity (820 mAh g^−1^) make it an attractive option for energy storage applications [13]. ZIBs utilize either a slightly acidic or nearly neutral aqueous electrolyte, enhancing both cycle safety and reversible capacity. The aqueous medium within ZIBs exhibits a higher ionic conductivity (1 S cm^−1^) compared to the 1 to 10 mS cm^−1^ found in organic electrolytes, thereby facilitating rapid ion migration and potentially faster charging and discharging cycles. Consequently, AZIBs present a viable and environmentally friendly alternative to LIBs, contributing to the advancement of sustainable battery technologies [14,15,16].

However, the development of aqueous zinc-ion batteries (AZIBs) is currently hindered by significant challenges, primarily due to the absence of cathode materials that can deliver high capacity while maintaining structural stability during Zn^2+^ accumulation [17,18,19,20]. Various cathode materials have been extensively investigated, including manganese (Mn)-based [21,22,23,24,25,26], vanadium (V)-based [27,28,29,30,31], and Prussian blue analogs [32,33]. Among these, vanadium-based materials are particularly notable for their adjustable and controllable layered architectures and their capacity to exist in multiple oxidation states, which have garnered significant scientific interest. Nevertheless, the intrinsic layered structure of vanadium-based materials frequently collapses during the repeated zinc ion insertion and de-insertion processes, leading to poor cyclic stability [34,35].

Given the large ionic radius and low mass of NH_4_^+^ [36,37,38], it could potentially serve as a structural “pillar” within the layers of vanadium-based materials, thereby facilitating Zn^2+^ insertion and de-insertion. In light of these considerations, the recent surge in research on various ammonium vanadium oxides is noteworthy. These materials, increasingly considered for use in AZIBs cathode materials, have demonstrated promising performance. Examples include NH_4_V_4_O_10_ [39,40], (NH_4_)_2_V_4_O_9_ [41], and (NH_4_)_2_V_3_O_8_ [42]. However, the mechanism by which NH_4_^+^ enhances the electrochemical performance of these materials has not yet been fully elucidated.

Our density functional theory (DFT) calculations suggest that the introduction of NH_4_^+^ into the V_6_O_16_ lattice primarily facilitates charge transfer between Zn^2+^ and NH_4_^+^ ions, significantly reducing the direct electrostatic interactions between Zn^2+^ and the lattice framework of the cathode material. NH_4_^+^ acts effectively as a “lubricant”, attenuating the intensity of electrostatic fields experienced by Zn^2+^ ions, thereby promoting smoother ion diffusion. Based on these DFT predictions, we synthesized (NH_4_)_2_V_6_O_16_ (NHVO) using a one-step hydrothermal method. The experimental results and DFT calculations are in agreement, showing that NHVO cathodes exhibit remarkably stable electrochemical performance, along with high specific capacity and excellent cycle stability and life. After 100 cycles at 0.2 A g^−1^, the specific capacity remains at 208.3 mAh g^−1^, and after 2000 cycles at 5 A g^−1^, a capacity retention of 90% is maintained with 141.8 mAh g^−1^. These findings provide a clear direction for the design of cathode materials for AZIBs.

## 2. Results and Discussion

### 2.1. DFT Calculations

**Figure 1:**
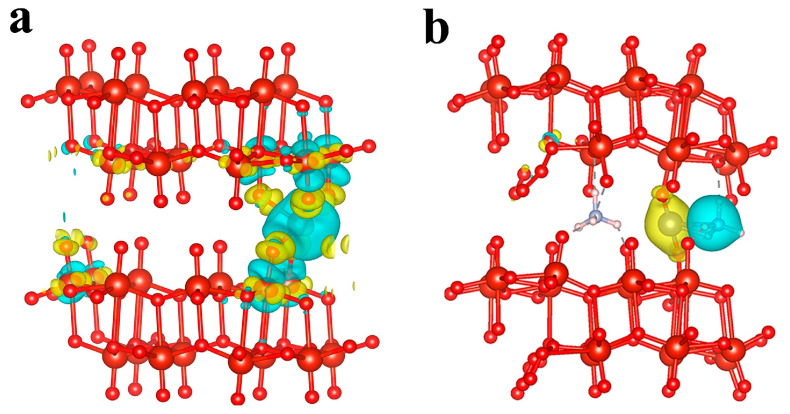
Optimized structure of and charge density difference in (**a**) Zn-VO and (**b**) Zn-NHVO.

We first employed DFT calculations to explore the effect of NH_4_^+^ incorporation on the electrochemical properties of vanadium-based cathode materials, focusing particularly on the charge transfer interactions involving Zn^2+^ ions. Two computational models were meticulously optimized: Zn^2+^ incorporated into V_6_O_16_ (referred to as Zn-VO) and Zn^2+^ incorporated into (NH_4_)_2_V_6_O_16_ (referred to as Zn-NHVO), with the results depicted in Figure 1a,b.

The charge density difference calculations for these models provide crucial insights into the electrostatic interactions within the cathode materials. In the Zn-VO model, a significant charge transfer is observed between Zn^2+^ and the oxygen atoms in the V-O bonds (as shown in Figure 1a). This charge transfer manifests as a reduction in charge density near Zn^2+^, depicted in blue, and an increase in surrounding regions, shown in yellow. The interaction leads to a strong electrostatic attraction between Zn^2+^ and the lattice oxygen, which impedes the diffusion of Zn^2+^. This strong interaction not only hinders the mobility of Zn^2+^ within the cathode but also contributes to structural instability during battery cycling, potentially leading to material degradation and collapse.

Conversely, in the Zn-NHVO model, the introduction of NH_4_^+^ modifies the interaction landscape dramatically. Here, the primary charge transfer occurs between Zn^2+^ and NH_4_^+^ ions, significantly reducing the direct electrostatic interactions between Zn^2+^ and the lattice framework of the cathode material. The presence of NH_4_^+^ mitigates the intensity of the electrostatic fields experienced by Zn^2+^ ions, thereby facilitating smoother ion diffusion, as evidenced by the yellow regions in Figure 1b, which denote an increase in charge density. This alteration suggests that NH_4_^+^ acts effectively as a ”lubricant”, enhancing the mobility of Zn^2+^ through the cathode material and improving the structural stability during the electrochemical cycling process.

These findings underscore the potential of NH_4_^+^ as a strategic additive for enhancing the performance of vanadium-based cathode materials. By altering the charge distribution and reducing detrimental interactions within the cathode, NH_4_^+^ inclusion leads to improved ion diffusion capabilities and increased material stability.

### 2.2. Morphological Characterization

**Figure 2:**
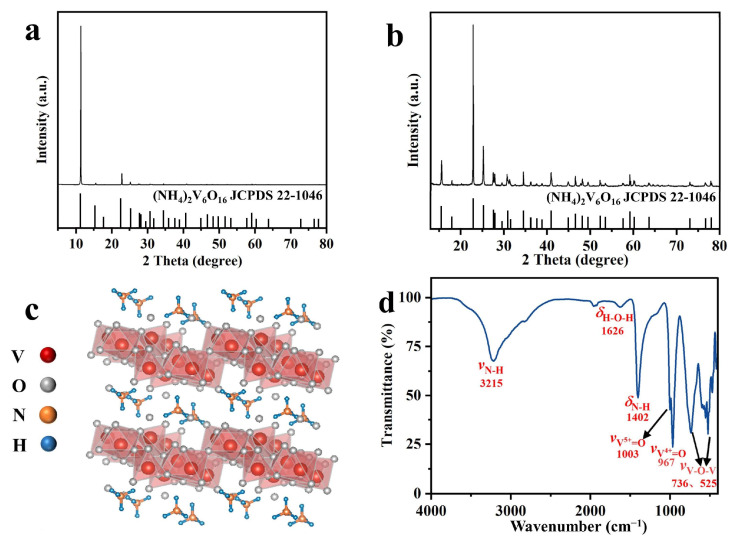
(**a**) XRD patterns of NHVO at 5–80°; (**b**) XRD patterns of NHVO at 15–80°; (**c**) Crystal structure of NHVO; (**d**) FTIR spectrum of NHVO.

To verify the results from the DFT calculations, we synthesized NHVO using a one-step hydrothermal method and characterized it using X-ray Powder Diffraction (XRD) and Fourier Transform Infrared Spectroscopy (FTIR). The XRD patterns, shown in Figure 2a,b, predominantly exhibit diffraction peaks that align well with those of (NH_4_)_2_V_6_O_16_ (JCPDS No. 22-1046, space group: P2_1_/m), indicating the successful synthesis of the material.

The crystal structure of NHVO, depicted in Figure 2c, consists of alternating chains of VO_5_ pyramids and VO_6_ octahedra, which are interconnected through shared vertices and edges. This arrangement supports a layered structure, with ammonium ions acting as ‘pillars’ that stabilize the framework.

Further confirmation of the synthesized NHVO’s structural integrity was provided through FTIR analysis, represented in Figure 2d. The observed peaks at 736 cm^−1^ and 525 cm^−1^ are attributed to the asymmetric and symmetric stretching vibrations of V–O bonds, respectively. Peaks at 967 cm^−1^ and 1003 cm^−1^ correspond to the stretching vibrations of V^4+^=O and V^5+^=O in the VO_5_ pyramids and VO_6_ octahedra, indicating the presence of both vanadium oxidation states, which are critical to the material’s functionality. Additionally, the peaks at 3215 cm^−1^ and 1402 cm^−1^ are identified as the asymmetric stretching and symmetric bending vibrations of N–H bonds, further confirming the successful incorporation of ammonium ions within the layered structure.

**Figure 3:**
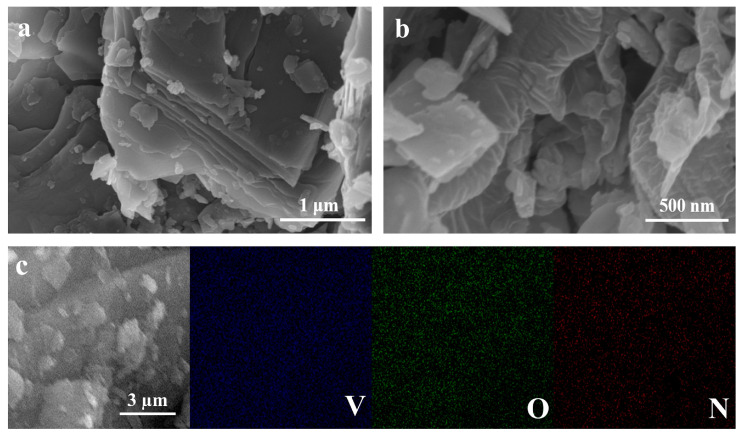
(**a**,**b**) SEM patterns of NHVO; (**c**) EDS mapping of NHVO.

The scanning electron microscopy (SEM) images (Figure 3a,b) of NHVO reveal its unique surface morphology characterized by uneven, overlapping nanosheets with a distinctly wrinkled texture. This morphology is particularly advantageous for cathode materials in AZIBs, as the high surface area facilitates Zn^2+^ intercalation and deintercalation, enhancing the electrochemical performance and ion diffusion kinetics. The wrinkled surfaces can potentially increase the electrode–electrolyte contact area, promoting better ion transfer and accessibility, which are critical for high-rate performance in batteries. Energy-dispersive spectroscopy (EDS) mapping (Figure 3c) complements the SEM analysis by confirming the homogeneous distribution of nitrogen (N), V, and oxygen (O) within the nanosheets. This uniformity in the elemental composition ensures consistent electrochemical behavior across the electrode, which is essential for achieving stable cycling performance in AZIBs.

**Figure 4:**
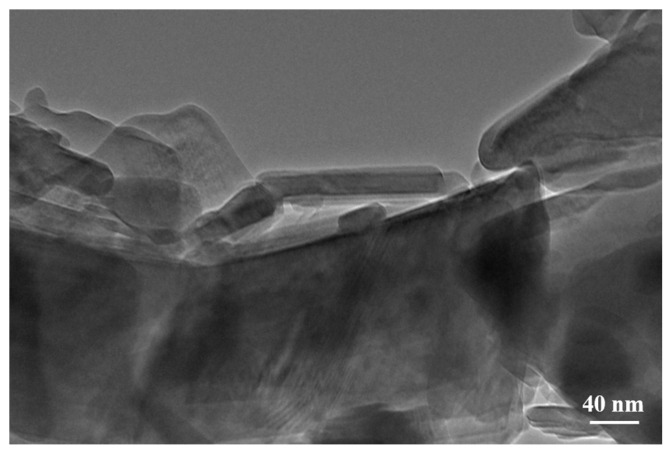
TEM image of NHVO.

Transmission electron microscopy (TEM) images (Figure 4) provide a closer look at the overlapping nanosheet morphology, further verifying the nanoscale features observed in the SEM images. The TEM analysis supports the structural details by showcasing the thin, layered nature of the nanosheets, which is ideal for facilitating short diffusion paths for zinc-ions. This structural confirmation through TEM indicates a well-synthesized material with characteristics that are supportive of its application as a cathode material in AZIBs.

### 2.3. Electrochemical Properties Characterization

The electrochemical performance of NHVO as a cathode material in AZIBs was rigorously evaluated through small current cycle performance tests, rate capability tests, and high current cycle tests. Vanadium-based compounds often exhibit poor cycle stability under low current densities due to the more thorough and slower reactions occurring on the electrode, which challenge the structural integrity of the material. Remarkably, NHVO demonstrates a unique behavior under such conditions.

**Figure 5:**
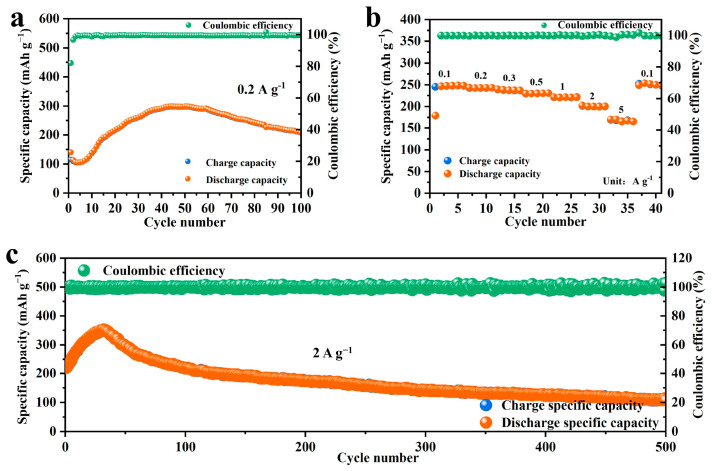
(**a**) The cycling performance of NHVO at a current density of 0.2 A g^−1^; (**b**) The rate performance of NHVO; (**c**) The cycling performance of NHVO at a current density of 2 A g^−1^.

Initially, at a low current density of 0.2 A g^−1^, NHVO displayed a modest capacity. However, as cycling progressed, the capacity gradually increased, indicating an activation process and stabilization of the electrode material. This was clearly demonstrated in the test results, where NHVO exhibited excellent cycling stability and Coulombic efficiency with no capacity fade over 100 cycles, as shown in Figure 5a. This suggests that NHVO, unlike typical vanadium-based materials, maintains structural integrity even under slow, exhaustive reaction conditions, which is critical for long-term applications in batteries.

Upon the activation of the electrode material at low currents, rate capability tests were conducted at varied current densities of 0.1, 0.2, 0.3, 0.5, 1, 2, and 5 A g^−1^ (Figure 5b). The specific capacities observed were 246.8, 242.6, 239.7, 229.8, 221.3, 202.2, and 170 mAh g^−1^, respectively. Notably, when the current density was reverted to 0.1 A g^−1^, the capacity recovered to 253.7 mAh g^−1^, highlighting the excellent reversibility and structural resilience of NHVO. These results, consistent with DFT calculations, confirm the predictive accuracy of DFT in assessing the structural stability and electrochemical behavior of NHVO.

Furthermore, NHVO demonstrated robust performance under high-current-density tests. After full activation at high currents, NHVO sustained a capacity of 157.1 mAh g^−1^ over 500 cycles at a current density of 2 A g^−1^, with a Coulombic efficiency approaching 100%, as depicted in Figure 5c. This performance underlines the feasibility of NHVO as a cathode material for AZIBs, showcasing its capability to handle significant electrochemical stresses without substantial degradation.

Moreover, as depicted in Figure 5a,c, the capacity of the NHVO cathode material exhibits an initial increase up to the 40th cycle at a current density of 0.2 A g^−1^, subsequently followed by a decline. The initial capacity enhancement from 0 to 40 cycles can be primarily attributed to the activation of the NHVO cathode material. During these initial cycles, electrochemical activation facilitates the enhancement of active site accessibility and improves the wettability of the electrode, thereby temporarily augmenting the capacity. Beyond the 40th cycle, the capacity progressively diminishes. This reduction can be principally ascribed to the dissolution of active material into the electrolyte. Furthermore, the repetitive intercalation and deintercalation of Zn^2+^ ions induce volumetric changes in the cathode material, which in turn generate mechanical stress and lead to a loss of electrical contact within the electrode structure.

**Figure 6:**
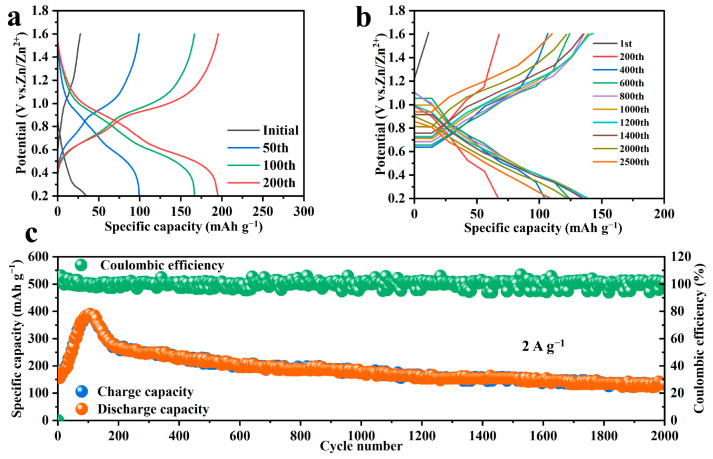
(**a**) The galvanostatic charge-discharge curves of NHVO at the current density of 0.2 A g^−1^ for the first cycle, the 50th cycle, the 100th cycle and the 200th cycle; (**b**) Galvanostatic charge-discharge curves of NHVO at a current density of 5 A g^−1^ for 2000 cycles; (**c**) Cycling performance of NHVO at a current density of 5 A g^−1^.

Galvanostatic charge–discharge tests were conducted on NHVO within a voltage range of 0.2–1.6 V at a constant current density of 0.2 A g^−1^, as depicted in Figure 6a. Initially, the discharge capacity was observed at 34.5 mAh g^−1^, followed by a charge capacity of 25.7 mAh g^−1^. With successive cycles, the electrode demonstrated gradual activation, leading to an increase in both discharge and charge capacities along with the formation of extended and stable electrochemical plateaus. Notably, the discharge profiles displayed significant plateaus at 0.9 V and 0.6 V. This behavior underlines the excellent electrochemical reversibility of NHVO, with average discharge and charge capacities stabilizing around 200.1 mAh g^−1^ and 200.3 mAh g^−1^ respectively, as illustrated in Figure 6a.

Further testing at a high current density of 5 A g^−1^ over 2000 cycles, as depicted in Figure 6b, showcased remarkable cycle stability and consistency in the charge–discharge profiles. This sustained performance, coupled with a high Coulombic efficiency of 99.9%, indicates that the (de)intercalation of Zn^2+^ ions within NHVO is highly reversible. These findings reinforce the predictions made by DFT calculations, highlighting the material’s robustness and efficiency under demanding conditions.

During the long cycling test at 5 A g^−1^, although the initial specific capacity was relatively low, it stabilized significantly upon full activation, maintaining a capacity of 141.8 mAh g^−1^. After 2000 cycles, the capacity retention was impressively high at 94%, as shown in Figure 6c. This endurance, especially under high current conditions, suggests that NHVO possesses exceptional structural stability and can endure the stresses of rapid charge and discharge processes without significant degradation.

**Figure 7:**
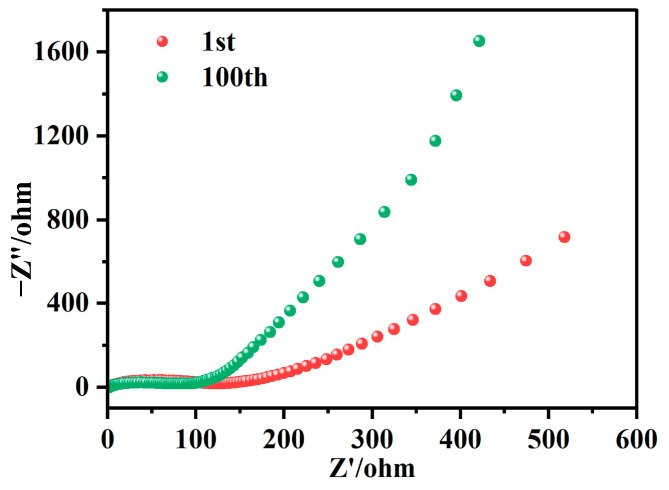
EIS profiles of the 1st and 100th laps of NHVO.

Electrochemical impedance spectroscopy (EIS) was employed to characterize the impedance properties of NHVO over the frequency range of 0.01 Hz to 100,000 Hz. As depicted in Figure 7, the impedance spectra for NHVO are composed of a semicircle in the low-frequency region and a linear “tail” in the high-frequency region. These features can be interpreted as indicative of two primary electrochemical processes: charge transfer and mass transfer. The charge transfer resistance (*R*_ct_) and the Warburg impedance (*Z*_w_), which abstract these processes, are key parameters in analyzing the electrode dynamics.

In the EIS spectrum, the semicircle at higher frequencies corresponds to *R*_ct_, whereas the linear part at lower frequencies is associated with *Z*_w_, which models the diffusion of ions within the electrode material. In practical terms, the impedance due to Warburg effects is considered negligible at very low frequencies (close to zero impedance) and becomes significant at higher frequencies.

Initially, the EIS measurements revealed a relatively high *R*_ct_ for the NHVO cathode. This heightened initial resistance can be attributed to the pristine state of the electrode material, where electron conduction pathways are suboptimal, and the electrode/electrolyte interfaces are not fully established.

Upon subsequent cycling, particularly after 100 cycles at a low current regime, a notable reduction in *R*_ct_ was observed. This decrease suggests several advantageous modifications to the electrode’s microstructure and chemistry induced by the cycling process. First, the application of electrical stress during cycling promotes the formation of new, more efficient pathways for electron conduction [43]. Such pathways often arise through minor restructuring of the electrode material, which may include the formation of micro-cracks or other microstructural rearrangements that diminish barriers to electron mobility.

Moreover, the repeated intercalation and deintercalation of ions during cycling contribute to a more activated state of the electrode surface. This electrochemical activation aids in reducing polarization at the electrode/electrolyte interface, thereby enhancing the kinetics of the involved electrochemical reactions. These alterations generally coincide with an increase in electrical conductivity and a reduction in overall resistance to charge transfer within the electrode material.

Additionally, the cycling process facilitates the partial exfoliation of layered materials within the electrode, exposing fresher, more reactive surfaces that enhance electrochemical reactivity and charge transfer efficiency. This characteristic of electrode behavior under cycling conditions is pivotal for applications demanding high durability and efficiency over prolonged usage, such as in batteries and supercapacitors.

In summary, the observed post-cycling decrease in *R*_ct_ is primarily attributable to the formation of efficient electron conduction pathways and the activation of the electrode material. These changes collectively improve the electrochemical performance of the NHVO cathode.

### 2.4. Storage Mechanism of Zn^2+^

**Figure 8:**
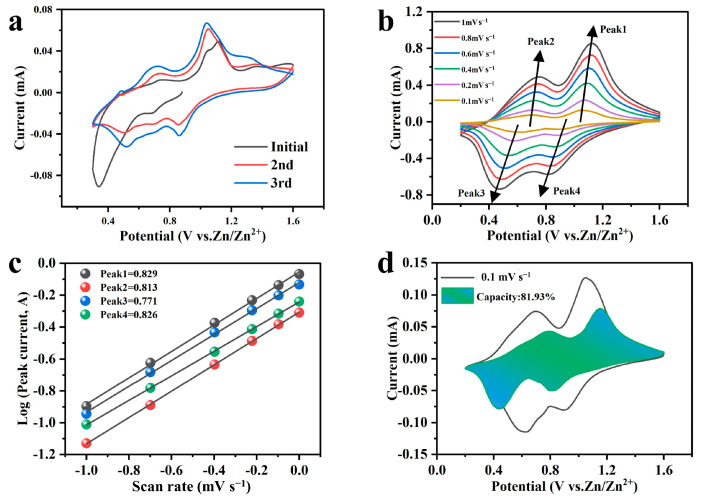
(**a**) CV curves of NHVO in the initial three cycles; (**b**) Cyclic voltammetry curves of NHVO at various scan rates; (**c**) Log (*i*) vs. log (*v*) plots corresponding to the four peak currents in the CV curves; (**d**) Pseudocapacitance contribution of NHVO at a scan rate of 0.1 mV s^−1^.

Cyclic voltammetry (CV) tests were conducted on NHVO to elucidate the Zn^2+^ storage mechanism within this cathode material for aqueous AZIBs. The tests were performed at a scan rate of 0.1 mV s^−1^ across a voltage range of 0.2–1.6 V (relative to Zn^2+^/Zn), as shown in Figure 8a. The initial three cycles revealed two distinct pairs of redox peaks around 0.52/0.71 V and 0.85/1.05 V, indicative of the intercalation/deintercalation of Zn^2+^ ions within the NHVO host material. These observations suggest a multi-step reaction mechanism, which is typical for materials where multiple valence states facilitate the storage process.

Notably, a slight shift in the redox peaks was observed from the very first cycle, suggesting an initial activation of the cathode. This transition reflects changes in the electrode’s surface properties or the formation of new active sites, enhancing the electrochemical reactivity. The overlapping nature of the subsequent cycles indicates good reversibility in the Zn^2+^ intercalation/deintercalation process within the layers of NHVO. This reversible behavior is crucial for the long-term stability and efficiency of the battery.

The presence of well-defined redox peaks also underscores the potential for phase transitions within the NHVO structure, facilitated by the cycling process. The consistent appearance of these peaks in subsequent cycles suggests that the NHVO structure accommodates the Zn^2+^ without significant degradation or structural collapse. This stability in the redox behavior across cycles highlights the robustness of NHVO as a cathode material, capable of sustaining repeated electrochemical processes essential for high-performance zinc-ion batteries.

The CV curves of NHVO electrodes at different scan rates, depicted in Figure 8b, provide crucial insights into the electrochemical behavior and Zn^2+^ storage mechanisms of the NHVO cathode material in aqueous zinc-ion batteries. Analyzing the relationship between the current (*i*) and scan rate (*v*) offers a method for qualitatively assessing the contributions of capacitive effects using Equations (1) and (2):

*i* = *av^b^* (1)

log(*i*) = log(*a*) + *b*log(*v*) (2)

Here, the exponent *b* is a pivotal factor that distinguishes the controlling electrochemical processes. Values of *b* close to 0.5 typically suggest a predominance of ionic diffusion, whereas values nearing 1.0 indicate control by capacitive behavior. The *b* values calculated from the peak currents, specifically for peaks 1–4, were found to be 0.82, 0.81, 0.77, and 0.82, respectively (see Figure 8c). These values reveal that the electrochemical reactions are controlled by a combination of capacitive and diffusion behaviors.

Moreover, the respective currents at different scan rates can be described by Equation (3):

*i* = *k*_1_*v* + *k*_2_*v*^1/2^ (3)

**Figure 9:**
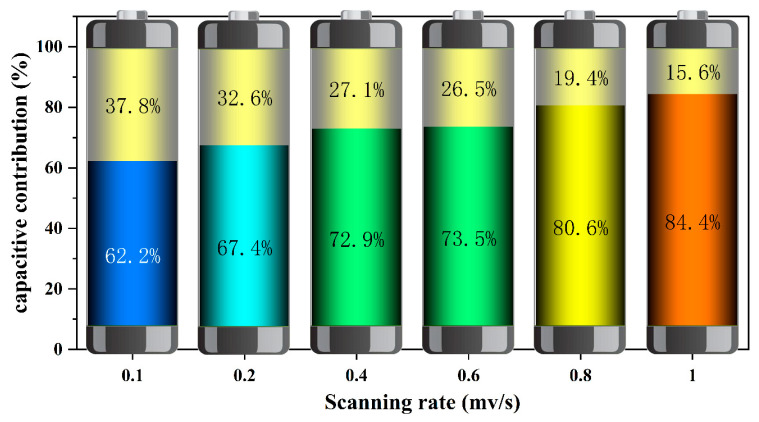
Pseudocapacitance contribution of NHVO at different scan rates.

In this model, *k*_1_*v* and *k*_2_*v*^1/2^ represent the contributions from capacitive processes and diffusion-controlled processes, respectively. For instance, in Figure 8d (at 0.1 mV s^−1^), the green area signifies the capacitive contribution, and the remainder of the CV curve indicates diffusion control. This depiction aligns with the pseudo-capacitive contributions calculated at various scan rates, as outlined in Figure 9, with values of 62%, 67%, 73%, 73.5%, 80.6%, and 84.4% at scan rates of 0.1, 0.2, 0.4, 0.6, 0.8, and 1 mV s^−1^ respectively. At lower scan rates, the electrochemical behavior is predominantly influenced by ionic diffusion, whereas at higher scan rates, capacitive processes increasingly dominate, playing a more crucial role in the electrochemistry of the NHVO electrodes.

**Figure 10:**
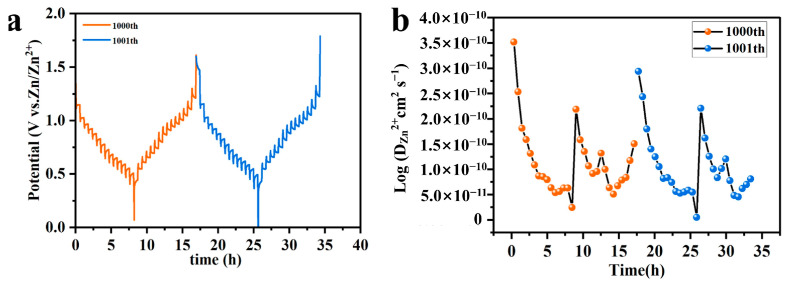
(**a**) GITT profiles of the Zn/NHVO battery; (**b**) The corresponding diffusivity coefficient for Zn^2+^ in the Zn/NHVO battery.

To further investigate the electrochemical reaction mechanisms within the Zn/NHVO battery, the galvanostatic intermittent titration technique (GITT) was employed to analyze the diffusion dynamics of Zn^2+^ ions after 1000 charge–discharge cycles, as depicted in Figure 10a. The GITT experiments were systematically conducted, involving a series of pulse applications followed by operation under a constant current and concluding with a relaxation period. A current density of 0.1 A g^−1^ was maintained throughout the experiments. The relaxation period was set to 30 min, and measurements were recorded at intervals of 10 s. The calculation is based on the following equation:

(4) $$D^{GITT}=\frac{4}{\pi \tau }{\left(\frac{m_{B}V_{M}}{M_{B}S}\right)}^{2}{\left(\frac{{\Delta E}_{S}}{\Delta E_{t}}\right)}^{2}$$

where *τ* represents the relaxation time, *m_B_* is the mass of active material, *V_M_* denotes the molar volume, *M_B_* is the molar mass, *S* is the surface area of the electrode, and Δ*E_S_* and Δ*E_t_* are the steady-state and transient potential changes, respectively. This analytical approach yielded a diffusion coefficient for Zn^2+^ ions ranging from 10^−11^ to 10^−10^ cm^2^ s^−1^. These values are significantly superior to those reported for several other vanadium-based cathode materials in AZIBs, underscoring the enhanced diffusion properties of the novel NHVO material.

The consistent observation of *D*_Zn2+_ values throughout the entire GITT cycle, shown in Figure 10b, confirms the robust and reliable diffusion characteristics of the NHVO material. A high diffusion coefficient is indicative of the material’s capacity to facilitate rapid Zn^2+^ ion transport, which is critical for achieving excellent rate performance. This feature is essential in applications requiring a high power output and efficient energy delivery, particularly under rapid charge and discharge conditions.

Furthermore, the preservation of high *D*_Zn2+_ values after 1000 cycles points to the structural stability and integrity of the NHVO material. This stability suggests that the NHVO electrode maintains effective ion pathways and a crystalline structure, despite undergoing repeated intercalation and deintercalation processes of Zn^2+^ ions. Such durability is crucial for the long-term usability and cycle life of zinc-ion batteries, reinforcing the potential of NHVO as a superior cathode material.

In addition, ex situ XRD and X-ray photoelectron spectroscopy (XPS) analyses were employed to probe the electrochemical mechanisms of NHVO. These techniques were utilized to examine the electrode materials cycled over a voltage range of 0.2 to 1.6 V for 100 cycles to ensure full activation. Following activation, the electrodes were analyzed at the discharged state of 0.2 V and the charged state of 1.6 V.

**Figure 11:**
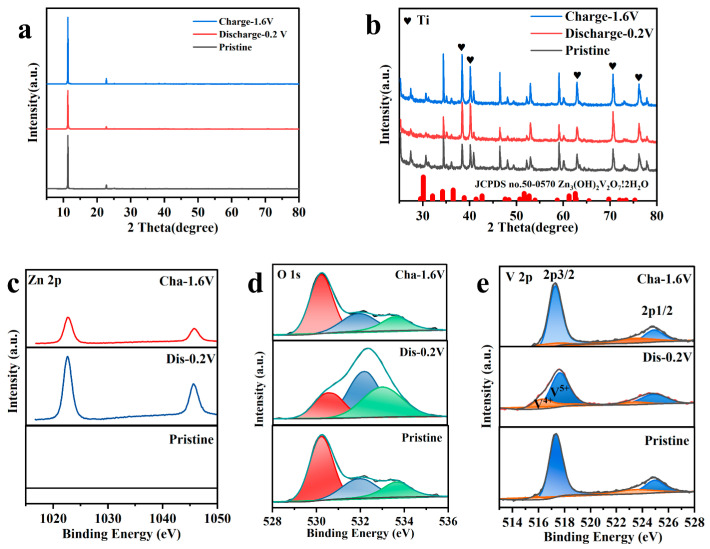
Ex situ XRD patterns of NHVO after 100 cycles at (**a**) 5–80° and (**b**) 25–80°; (**c**–**e**) Ex situ XPS patterns of NHVO.

The XRD patterns, displayed in Figure 11a,b, covering the 5–80° and 25–80° ranges respectively, show that the primary diffraction peaks maintain their positions across the cycling process. This observation implies that the crystal structure of NHVO remains relatively stable, with no significant phase transitions occurring during cycling. The stability in peak positions is indicative of the robustness of NHVO under operational conditions, highlighting its suitability for long-term applications in battery technology. During the discharge to 0.2 V, new peaks appear, as seen in Figure 11b, which correspond to the formation of an intermediate phase, identified as Zn_3_(OH)_2_V_2_O_7_·2H_2_O. The appearance of these peaks suggests the involvement of water molecules in the structural matrix of NHVO, which could be critical for the intercalation mechanism of Zn^2+^ ions.

Significantly, the peak at approximately 12°, corresponding to the (001) planes of NHVO, shifts slightly leftwards when discharged to 0.2 V, indicative of an increase in the interlayer spacing. This alteration can be attributed to the intercalation of Zn^2+^ ions, which enlarge the layer spacing to accommodate their size. Upon recharging to 1.6 V, the original peak position is restored, demonstrating the reversible nature of this intercalation process.

Figure 11c–e display the XPS patterns for Zn, V, and O elements in the NHVO electrodes at various stages: the pristine electrode, discharged to 0.2 V after three cycles, and recharged to 1.6 V. Initially, the pristine NHVO electrode displayed no detectable Zn signals, which is expected, as Zn is not a constituent of the virgin material. Upon discharging to 0.2 V, a prominent Zn 2p_3/2_ peak was observed at 1022.58 eV, indicating the intercalation of Zn^2+^ ions into the electrode matrix. This peak, however, diminished in intensity but remained visible at 1022.4 eV when the electrode was fully recharged to 1.6 V, suggesting some degree of irreversible Zn^2+^ intercalation during the electrode activation process.

For vanadium, the XPS spectra revealed binding energies at 516.2 eV and 517.8 eV, corresponding to the V2p_3/2_ peaks of V^4+^ and V^5+^ states, respectively. Notably, the proportion of V^4+^ increased after the third full discharge, a change attributed to the reductive reaction occurring at the cathode due to Zn^2+^ insertion. This proportion decreased upon full recharge, indicating a reversible oxidation–reduction process linked with the electrochemical cycling of the electrode.

The oxygen 1s spectrum for the NHVO electrode, which can be deconvoluted into three peaks at 530.18, 531.8, and 533.68 eV, corresponds to lattice oxygen in V=O bonds, hydroxyl groups (OH^−^), and water (H_2_O), respectively. The proportion of water-associated signals increased significantly during the discharge cycle, which benefits the electrochemical dynamics by providing the electrostatic shielding of metal ions, enhancing ion mobility and thus improving the kinetics of the electrochemical reactions and high-rate performance capabilities. The full reversibility of these peaks after charging indicates the good reversibility of the NHVO electrodes.

**Figure 12:**
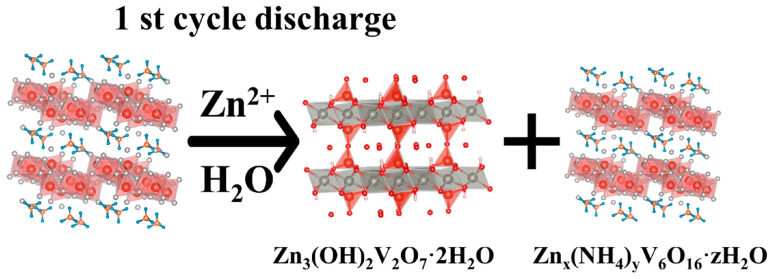
Schematic diagram of the Zn^2+^ storage mechanism of NHVO.

Figure 12 schematically illustrates the structural changes within the electrode during cycling. Initially, during the first discharge where Zn^2+^ irreversibly intercalates, Zn^2+^ and H_2_O incorporation into the NHVO interlayers results in an expansion of the adjacent VO layers. This leads to a partial transformation of NHVO into Zn_x_(NH_4_)_y_V_6_O_16_·zH_2_O and Zn_3_(OH)_2_V_2_O_7_·2H_2_O. In subsequent cycles, both phases coexist with reversible intercalation and deintercalation of Zn^2+^, exhibiting high reversibility consistent with the previous literature.

## 3. Materials and Methods

### 3.1. Calculation Method

DFT calculations were conducted using the Vienna Ab initio Simulation Package (VASP) [44]. The exchange–correlation effects were modeled with the Perdew–Burke–Ernzerhof (PBE) functional [45]. A plane-wave basis set was employed, with an energy cutoff set to 400 eV, to ensure adequate representation of the wavefunctions. The Projector Augmented Wave (PAW) method [46,47] was utilized for the treatment of core and valence electrons. Convergence criteria were stringently defined: the self-consistent field (SCF) iterations were set to converge at a threshold of 1 × 10^−5^ eV, and the force convergence was set at 0.01 eV Å^−1^. For the structural relaxations and total energy calculations, the Brillouin zone was sampled using a Γ-centered k-point mesh configured as 2 × 4 × 1.

### 3.2. Preparation of Material

NHVO was synthesized using a one-step hydrothermal method. Initially, 4 mmol of ammonium metavanadate was added to 25 mL of deionized water. The mixture was heated to 80 °C and stirred for 30 min using a jacketed magnetic heating stirrer to achieve complete dissolution. Subsequently, 6 M nitric acid (HNO_3_) was gradually added to the solution. The addition of HNO_3_ was stopped once the solution transitioned from a pale yellow to a clear orange–yellow color, after which stirring was continued for an additional hour.

Following this, the solution was transferred into a 50 mL polytetrafluoroethylene (PTFE) autoclave and maintained at 150 °C for 12 h to facilitate the reaction. After the reaction, the autoclave was allowed to cool to room temperature. The resulting product was orange crystalline flakes, which were then washed three times with deionized water and anhydrous ethanol until the wash effluent was neutral. The flakes were subsequently dried in a vacuum oven at 60 °C for 12 h. Given the good crystallinity of the dried samples, they were further processed by grinding thoroughly and then subjected to ultrasonic dispersion for one hour to ensure uniform particle size. The dispersed samples were then placed back into the vacuum oven and dried again at 60 °C for an additional 12 h.

### 3.3. Materials Characterization

The XRD measurements were performed with Cu Kα radiation using a Smart Lab SE system (Tokyo, Japan), which provided detailed insights into the crystalline structure of the materials. Morphological analyses were conducted using SEM and TEM to investigate the surface and internal structural features of the materials. SEM images were captured using a Hitachi SU8010 (Tokyo, Japan), which allowed for high-resolution visualization of the material surfaces. TEM analyses were performed with an FEI Talos F200X (Waltham, MA, USA), enabling detailed observation of the nanostructure and morphology at higher magnifications.

The elemental distribution within the synthesized materials was assessed using the EDS feature of the SEM. This technique facilitated the quantitative and qualitative analysis of the elemental composition at various points across the samples, providing insights into the uniformity and purity of the synthesized materials.

XPS was utilized to further investigate the elemental composition and monitor changes in the oxidation states of the elements involved. These analyses were carried out using a Thermo ESCALAB 250Xi (Waltham, MA, USA), which offered high-resolution spectral data for both powder samples and sliced electrode materials. This method was particularly valuable for understanding the electronic environment of the elements and tracking changes due to electrochemical processes.

Additionally, FTIR was employed to identify the functional groups present within the materials. The FTIR analyses were conducted using a VERTEX 70 system (Saarbrücken, Germany), which provided detailed information on the molecular bonding and structure through the absorption spectra, contributing to a comprehensive understanding of the chemical properties of the cathode materials.

### 3.4. Electrode Fabrication

The electrode is composed of prepared active material, acetylene black (conducting agent), and poly(1,1-difluoroethylene) (binder) at a ratio of 6:3:1. These components are ground thoroughly to achieve a uniform mixture. Subsequently, N-Methyl-2-pyrrolidone (NMP) is added dropwise to form a slurry, which is then evenly spread onto a 0.1 mm thick titanium foil and dried in a vacuum oven at 110 °C for 12 h. The electrode films are then cut into 10 mm diameter discs using a die cutter, with a loading of approximately 1.8 mg·cm^−2^. The coin cells are assembled using a CR2032-type case, employing a Zn metal sheet as the anode, a 3M zinc trifluoromethanesulfonate (Zn(CF_3_SO_3_)_2_) solution as the electrolyte, titanium foil as the current collector, and glass fiber as the separator. The assembly sequence is the anode casing, Zn metal anode, electrolyte, separator, electrolyte (Zn(CF_3_SO_3_)_2_), cathode (active material on titanium foil), and cathode casing, followed by hydraulic sealing using a battery crimping machine. The assembled batteries are left to rest for 6 h before use. All operations are conducted under ambient air conditions.

### 3.5. Electrochemical Measurements

CV analyses were performed utilizing a coin cell configuration with two electrodes on a CHI 760E electrochemical workstation (Shanghai Chenhua Apparatus Co., Shanghai, China). The cathode sheet, coated with the active material, served as the working electrode and was assessed across a voltage range from 0.2 V to 1.6 V (vs. Zn/Zn^2+^), at varying scan rates of 0.1, 0.2, 0.4, 0.6, 0.8, and 1 mV s^−1^). EIS was executed by applying a small-amplitude sinusoidal AC signal, facilitating the measurement of the system’s impedance. Analyses were conducted using equivalent circuit modeling on the CHI 760E, spanning frequencies from 0.01 Hz to 10,000 kHz, with a voltage amplitude of 5 mV. The GITT was employed to investigate the diffusion processes and the interplay between charge transfer and electrochemical reactions at the electrode surface. This technique included cycles of pulse application, constant current, and relaxation, enabling the determination of the chemical diffusion coefficient. The tests were conducted using the CT2001A Battery Test System from Wuhan LAND Electric Co. (Wuhan, China). Furthermore, electrochemical cycling and rate capability assessments were carried out at room temperature on coin cells within a voltage range of 0.2 V to 1.6 V, utilizing the same CT2001A system. These methodologies provided a comprehensive evaluation of the electrochemical properties of the electrodes examined.

## 4. Conclusions

In conclusion, the integration of DFT calculations and experimental validation in this study has successfully elucidated the role of NH_4_^+^ ions in enhancing the structural and electrochemical performance of vanadium oxide cathodes in AZIBs. DFT predictions confirmed that the incorporation of NH_4_^+^ into the V_6_O_16_ lattice facilitates charge transfer between Zn^2+^ and NH_4_^+^ ions, significantly reducing the electrostatic interactions that impede ion diffusion. This effect essentially serves as a structural lubricant, promoting smoother ion migration and enhanced cyclic stability. Experimentally, the synthesized (NH_4_)_2_V_6_O_16_ cathodes displayed consistent alignment with these theoretical predictions, demonstrating not only a high specific capacity but also remarkable stability over prolonged cycles. The observed maintenance of 90% capacity retention after 2000 cycles at 5 A g^−1^ showcases the potential of NH_4_^+^-enhanced cathode materials in improving the durability and efficiency of AZIBs. These findings offer a promising direction for the development of more robust and sustainable energy storage devices, contributing significantly to the field of advanced electrochemical systems.

## Data Availability

The data presented in this study are available on request from the corresponding author.
